# Supplementary material for: Causal Relationship between Aging and Anorexia Nervosa: A White-Matter-Microstructure-Mediated Mendelian Randomization Analysis
Source: Biomedicines. 2024 Aug 16;12(8):1874. doi: 10.3390/biomedicines12081874 (PMC11351342; doi:10.3390/biomedicines12081874)
Supplement: Supplementary file 1 [file biomedicines-12-01874-s001.zip › Supplementary Figure/Figure S4/Singe SNP plot of MR analysis for TL to ubm-b-1460.pdf]

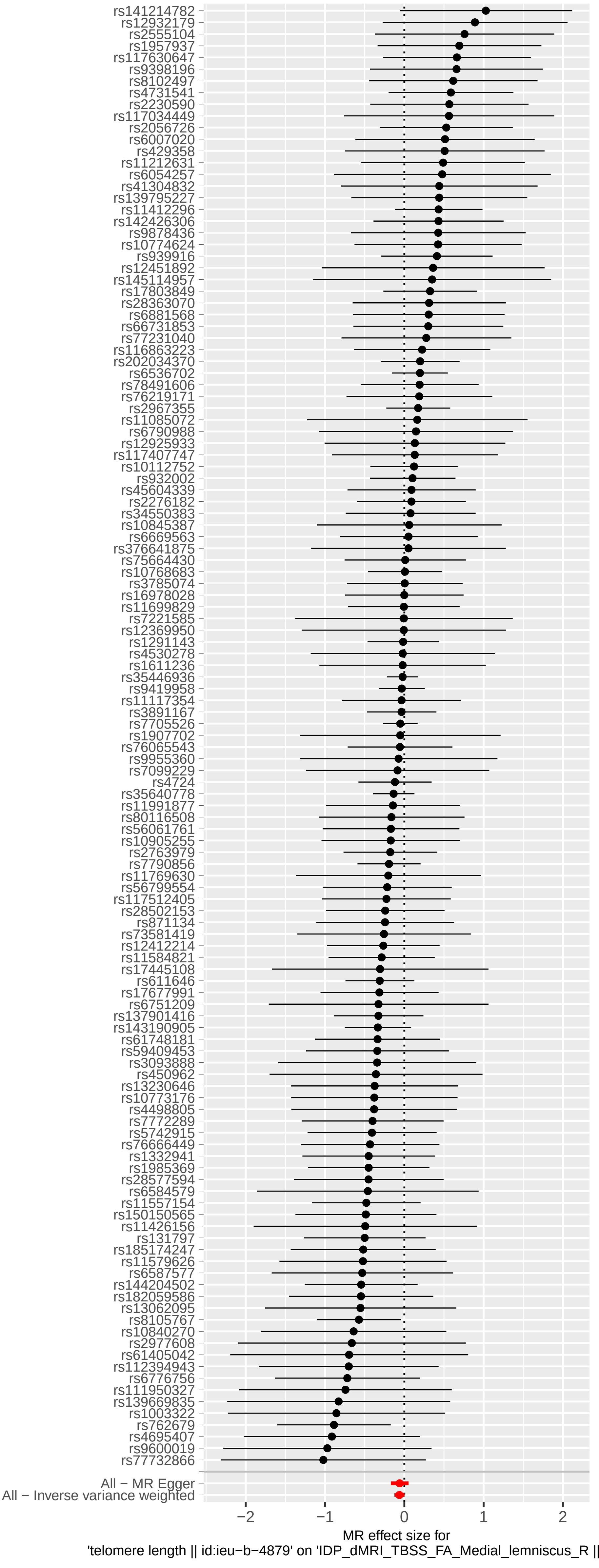
rs141214782
rs12932179
rs2555104
rs1957937
rs117630647
rs9398196
rs8102497
rs4731541
rs2230590
rs117034449
rs2056726
rs6007020
rs429358
rs11212631
rs6054257
rs41304832
rs139795227
rs11412296
rs142426306
rs9878436
rs10774624
rs939916
rs12451892
rs145114957
rs17803849
rs28363070
rs6881568
rs66731853
rs77231040
rs116863223
rs202034370
rs6536702
rs78491606
rs76219171
rs2967355
rs11085072
rs6790988
rs12925933
rs117407747
rs10112752
rs932002
rs45604339
rs2276182
rs34550383
rs10845387
rs6669563
rs376641875
rs75664430
rs10768683
rs3785074
rs16978028
rs11699829
rs7221585
rs12369950
rs1291143
rs4530278
rs1611236
rs35446936
rs9419958
rs11117354
rs3891167
rs7705526
rs1907702
rs76065543
rs9955360
rs7099229
rs4724
rs35640778
rs11991877
rs80116508
rs56061761
rs10905255
rs2763979
rs7790856
rs11769630
rs56799554
rs117512405
rs28502153
rs871134
rs73581419
rs12412214
rs11584821
rs17445108
rs611646
rs17677991
rs6751209
rs137901416
rs143190905
rs61748181
rs59409453
rs3093888
rs450962
rs13230646
rs10773176
rs4498805
rs7772289
rs5742915
rs76666449
rs1332941
rs1985369
rs28577594
rs6584579
rs11557154
rs150150565
rs11426156
rs131797
rs185174247
rs11579626
rs6587577
rs144204502
rs182059586
rs13062095
rs8105767
rs10840270
rs2977608
rs61405042
rs112394943
rs6776756
rs111950327
rs139669835
rs1003322
rs762679
rs4695407
rs9600019
rs77732866
All − MR Egger
All − Inverse variance weighted
−2
−1
0
1
2
MR effect size for
'telomere length || id:ieu−b−4879' on 'IDP_dMRI_TBSS_FA_Medial_lemniscus_R ||
